# Depot Dependent Effects of Dexamethasone on Gene Expression in Human Omental and Abdominal Subcutaneous Adipose Tissues from Obese Women

**DOI:** 10.1371/journal.pone.0167337

**Published:** 2016-12-22

**Authors:** R. Taylor Pickering, Mi-Jeong Lee, Kalypso Karastergiou, Adam Gower, Susan K. Fried

**Affiliations:** 1 Obesity Center, Department of Medicine, Boston University School of Medicine, Boston, MA, United States of America; 2 Clinical Translational Sciences Institute, Boston University, Boston, MA, United States of America; University of Barcelona, Faculty of Biology, SPAIN

## Abstract

Glucocorticoids promote fat accumulation in visceral compared to subcutaneous depots, but the molecular mechanisms involved remain poorly understood. To identify long-term changes in gene expression that are differentially sensitive or responsive to glucocorticoids in these depots, paired samples of human omental (Om) and abdominal subcutaneous (Abdsc) adipose tissues obtained from obese women during elective surgery were cultured with the glucocorticoid receptor agonist dexamethasone (Dex, 0, 1, 10, 25 and 1000 nM) for 7 days. Dex regulated 32% of the 19,741 genes on the array, while 53% differed by Depot and 2.5% exhibited a Depot*Dex concentration interaction. Gene set enrichment analysis showed Dex regulation of the expected metabolic and inflammatory pathways in both depots. Cluster analysis of the 460 transcripts that exhibited an interaction of Depot and Dex concentration revealed sets of mRNAs for which the responses to Dex differed in magnitude, sensitivity or direction between the two depots as well as mRNAs that responded to Dex only in one depot. These transcripts were also clearly depot different in fresh adipose tissue and are implicated in processes that could affect adipose tissue distribution or functions (e.g. adipogenesis, triacylglycerol synthesis and storage, insulin action). Elucidation of the mechanisms underlying the depot differences in the effect of Dex on the expression of specific genes and pathways that regulate adipose function may offer novel insights into understanding the biology of visceral adipose tissues and their links to metabolic health.

## Introduction

The mass of visceral fat, defined as those depots located within the abdominal cavity and associated with digestive organs (i.e. omental and mesenteric), is associated with risk for type 2 diabetes and cardiovascular disease in both men and women [1]. Glucocorticoids (GCs) promote the preferential accumulation of fat in visceral depots as clearly observed in Cushing’s syndrome [2–4]. Depot differences in rates of triacylglycerol (TAG) turnover, inflammation and adipocyte cellularity are well documented in humans and mouse models [5–7], but the mechanisms that underlie depot-dependent variations in GC action and mechanisms that link the size of this depot to systemic metabolic dysfunction remain incompletely understood.

GCs integrate a wide variety of regulatory signals that control cell proliferation, metabolism, and inflammation [8]. The activity of the GC receptor (GR) is cell-type, gene and dose dependent, and regulated by multiple signals [9, 10]. Adipose tissue includes multiple cell types, including preadipocytes, endothelial cells, immune cells and adipocytes, all of which are targeted by GCs [11]. Thus, in addition to direct GC actions in each cell type, paracrine and endocrine interactions likely contribute to depot differences in GC actions on gene expression and thereby tissue function. Although cell culture models provide invaluable mechanistic information on each cell type, the cellular composition of different depots varies and the composition of its extracellular matrix (ECM) likely also contributes to the depot differences in GC actions [12]. Thus, unraveling the molecular details of crosstalk among cell types and pathways in intact adipose tissue is complex. The advantage of organ culture in this context is that this system provides a physiologically relevant three dimensional context for the analysis of human adipose tissue hormone action and comparison of depot differences.

To gain an integrated picture of mechanisms by which GCs modulate depot-dependent function, we chose to use an organ culture system in which the expression of key adipocyte genes (e.g. *ADIPOQ*, *LEP*, *GLUT4*, *LPL*) is synergistically upregulated by GCs and insulin, and maintained at initial levels for at least 7 days [13, 14]. Our previous studies addressed global effects of GCs on the adipose transcriptome in organ cultures of the two major central adipose depots in humans, visceral (omental, Om) and abdominal subcutaneous (Abdsc) using a 12K microarray [14]. These studies tested only one concentration of the type II GR agonist Dex (25 nM), added in the presence of 7 nM insulin [14]. Dex regulated ~20% of the adipose expressed genes and many genes and pathways, such as those that promote TAG synthesis and mediate insulin action, were affected similarly in both Om and Abdsc, but the magnitude of the effects was often depot-dependent [14]. A limitation of this prior study was that Dex effects are highly concentration-dependent [9], and we have previously documented lower sensitivity to submaximally-stimulating concentrations of Dex on LPL and leptin gene expression [13, 15].

GR interacts with co-activators or co-repressors to exert complex effects on transcriptional networks. Mechanisms by which GR interacts directly with their binding sites that selectively enhance/repress the transcription of clusters of genes are rapidly emerging from studies of cell culture models [9, 16, 17]. Toward the long-term goal of understanding mechanisms by which GCs differentially modulate gene expression in the complex microenvironments of human visceral and subcutaneous adipose tissues, the two main goals of the current study were: 1) to identify transcripts that are differentially sensitive to a range of Dex concentrations (0, 1, 10, 25, 1000 nM) in Om and Abdsc, and 2) to identify GC-regulated mRNAs that may play unanticipated roles in depot-dependent adipose biology, i.e. they were mainly expressed only in one depot and were responsive to Dex in that depot. We used Dex for the current mechanistic study because it is a specific GR agonist, and unlike cortisol, it cannot be inactivated or interact with the mineralocorticoid receptor (MR). To this end, we used Affymetrix Human Gene 1.0 ST microarrays that represent nearly all ~20,000 human genes and a more physiologically relevant concentration of insulin (0.7 nM) compared to our prior study [14].

## Materials and Methods

### Sample collection

Adipose tissue was sampled from elective surgeries on volunteers free of diabetes, cancer and inflammatory diseases by medical record, and not taking any medications that could affect metabolism, as previously described [15]. Table 1 shows the characteristics of subjects whose tissue was used for microarray and follow-up studies. All studies were conducted under Institutional Review Board approved protocols at the Boston Medical Center. All subjects signed informed consent.

**Table 1:** Characteristics of subjects used in microarray and subsequent studies.

Age, yr	Sex	Surgery	Chol	TAG	HbA1c (%)	Race	BMI, kg/m^2^
Subjects used for microarray							
48	F	TAH abdominal	N/A	N/A	5.2	AA	54
54	F	gastric bypass	151	80	N/A	AA	41
28	F	gastric bypass	196	146	5.9	H	42.5
34	F	gastric bypass	136	79	5.5	H	54
30	F	gastric bypass	177	37	5.5	H	36
Additional Subjects used for qPCR							
36	M	gastric bypass	226	210	5.3	H	39
23	F	gastric bypass	171	80	4.5	H	39

AA- African American, H-Hispanic; Chol-Serum Cholesterol (mg/dl), serum TAG (mg/dl), HbA1c (Hemoglobin A1c)

### Tissue processing

Immediately after excision, a small aliquot of tissue (~200 mg) was quick frozen in liquid nitrogen. The rest was carried to the laboratory in room temperature Medium 199, minced into 5–10 mg fragments, quickly rinsed in 0.9% saline, and ~300–400 mg were placed in organ culture in 15 ml of Medium 199 supplemented with 0.7 nM insulin plus 0, 1, 10, 25 or 1000 nM Dex for 7d. Cultures were re-fed every other day and the day prior to harvest on d7 [18].

### Gene expression

Total RNA was isolated with Trizol reagent (Life Technologies, Carlsbad, CA), cleaned with RNeasy MiniElute Cleanup Kit (Qiagen, Germantown, MD) and used for microarrays and quantitative PCR (qPCR). Depot differences in mRNA levels in quick frozen tissue were verified by qPCR. Total RNA was reverse transcribed using Transcriptor First-Strand cDNA synthesis kit (Roche, Indianapolis, IN) and qPCR was performed on a LightCycler 480 II (Roche) with commercially available TaqMan probes (Life Technologies). Cyclophilin A (PPIA) was used as a reference gene, and relative expression levels were calculated.

### Microarrays

Human Gene 1.0 ST arrays were used to profile gene expression in 3 independent paired samples of Om and Abdsc, after culture with Dex 0, 1, 10, and 1000 nM. Two samples were pooled from two different donors with similar characteristics and the magnitude of the Dex effect to increase GILZ and decrease IL-6 expression (by qPCR), and one sample represented a single donor (Table 1). Array results (log2-transformed) were normalized together using the Robust Multiarray Average algorithm and a Chip Definition File that maps the probes on the array to unique Entrez Gene identifiers.

### Gene set enrichment analysis (GSEA)

To determine biological pathways regulated by each concentration of Dex in each depot, we calculated the fold change for the normalized values of gene expression in Om and Abdsc depot at each Dex concentration as compared to the control (0 Dex). The average fold change among subjects for each gene for each depot-dose pair was used for GSEA Preranked analysis (Ver. 2.1.0, Broad Institute [19, 20]). KEGG, Reactome, Biocarta and PID databases were queried. Because there was substantial overlap for significantly enriched pathways and gene-lists, only KEGG results are presented. Analyses based on data ranked by the T-value for the paired T-test yielded similar results (not shown). Significantly up and down regulated KEGG pathways (FDRq values < 0.05) are listed in S2A–S2C Table.

### Statistical analyses

A linear mixed-effects (LME) model was used. Depot (Om vs. Abdsc) and concentration of Dex were treated as "fixed" independent variables and donor as a "random" independent variable. The interaction of Depot and Dex concentration ([Dex]) was included in the model. [Dex] was modeled as a categorical, unordered variable so as not to exclude genes with a nonlinear dose response. The interaction effect measures whether dose effect on the expression of a given gene differs between the depots. For example, the concentration of Dex that consistently affects a given gene or the magnitude of the effect is dependent on depot. After calculation of p values for each term for each gene, the Benjamini-Hochberg False Discovery Rate (FDR) correction was used to obtain FDR-corrected p values (a.k.a. FDRq values).

A cluster analysis was conducted on a subset of 460 genes with a FDRq less than 0.05 for the interaction of Depot and [Dex] that also showed an effect size over 1.2 fold up or down in at least one concentration of Dex in one depot, and expression values greater than a cutoff of 20 (out of a maximum ~6000) arbitrary units. The mean values for expression of these at each Dex concentration were scaled across both depots ($\frac{X-Avg}{SD}$). Cluster analysis was performed with JMP 10 software (using settings of complete, 1 or 2 way cluster, scaled).

Expression levels of selected genes of interest were verified by qPCR using 7 paired Om and Abdsc samples. Data are normalized by PPIA gene expression (2^-ΔCT^) and presented as the mean and SEM of relative abundance. All values were log-transformed prior to statistical analysis. The effect of Dex within a depot was tested with a one way repeated measures ANOVA (on dose), with post-hoc paired t-tests as indicated, within each depot. Paired t-tests were used to compare values in Abdsc and Om depots within subjects.

## Results

Using a linear mixed modeling approach and conservative cutoff of FDRq < 0.05, 6,344 out of 19,741 transcripts on the array (32%) were significantly regulated by Dex, as indicated by a significant main effect. 10,424 transcripts (53%) showed a Depot effect and 513 (2.5%) showed a Depot*[Dex] interaction. Gene lists, average expression values and FDRq values for the LME analysis are provided in S1 Table. To identify transcripts that are depot-independently regulated by Dex, expression levels at each Dex concentration in each depot were pre-ranked by fold-change for statistical analysis. If the FDRq for the Dex and Depot effect in the LME was significant (FDRq <0.05), and there was no Depot × [Dex] interaction, they were considered depot independently regulated by Dex. A significant [Dex]*Depot interaction indicates that the Dex effect varied by Depot. Similar results were obtained when genes were ranked by T-values for the Dex effect compared to baseline (not shown).

### Pathways that were regulated by Dex in a depot independent manner

*Dex upregulated pathways*: S2A Table lists the KEGG pathways identified by GSEA as upregulated in both depots (FDRq < 0.05 in both) at each concentration of Dex. As expected from our previous work [14], insulin signaling as well as metabolic pathways involved in fatty acid (FA), amino acid, and carbohydrate degradation/oxidation through pyruvate and the Krebs cycle (glycolysis, pyruvate metabolism pathway) were upregulated by Dex in both depots. Also, as expected, lipid metabolism-related and signaling pathways upregulated by Dex similarly in both depots included FA metabolism, peroxisome, biosynthesis of unsaturated FAs, glycerophospholipid metabolism and glycerolipid metabolism. The gluconeogenesis pathway was also upregulated; this list included *PCK1* which is of importance in adipocytes because it functions as a glyceroneogenic enzyme that regulates esterification of FA [21].

A key mediator of changes in metabolic gene expression, the PPAR signaling pathway was significantly enriched by exposure to Dex in both Om and Abdsc. The fold changes in these genes tended to be higher in Om, but the Depot × Dex interaction term was not significant. Expression levels at all concentrations of Dex were higher in Abdsc; *PCK1*, *LPL*, *ACADL*, *PLIN1*, *ME1*, *NR1H3* (also known as *LXRA*) and *ACSL1* contributed most highly to the enrichment of this pathway. Also of interest for its known role in adipocyte function [22], the KEGG retinol pathway was also upregulated by Dex in both depots. *ADH1B*, *ADH1A*, and *ALDH1A1* were at the leading edge of this list in both depots; *DGAT1*, a key gene in the regulation of TAG synthesis, also contributed to the enrichment score for this pathway.

*Dex downregulated pathways*: The most downregulated KEGG pathways common to Om and Abdsc depots were inflammatory and immune pathways; cytokine-cytokine receptor interaction, chemokine signaling, intestinal immune network for IgA production, leukocyte transendothelial migration, antigen processing and presentation and Toll-like receptor signaling. Dex also downregulated TGFβ as well as JAK-STAT signaling pathways in both depots. Pathways related to ECM including ECM receptor interaction, cell adhesion molecules, focal adhesion, and axon guidance were also downregulated by Dex. A number of transcripts on these lists with the highest negative enrichments have been implicated in adipose tissue inflammation and ECM remodeling, for example, Dex markedly downregulated *TNC* [23], which topped the ECM receptor interaction list in both depots (normalized enrichment score (NES) -5.0 in Abdsc and -5.7 in Om at 10 nM Dex, both p<0.0001). S2B Table lists the KEGG pathways identified as significantly downregulated in both depots at each concentration of Dex (FDRq for main effect of Dex < 0.05).

### Depot-dependent effects of Dex in Om or Abdsc

Two pathways were clearly upregulated by Dex only in Om but not Abdsc, steroid biosynthesis, which is of interest from the point of view of cholesterol and cortisol synthesis within adipose tissue, and the pentose phosphate pathway (S2C Table). The latter is important in generating reducing equivalents for de novo FA synthesis and steroid synthesis (e.g. *H6PDH*).

Several downregulated pathways were clearly affected in Abdsc but not Om, depending on Dex concentration (S2C Table). Transcripts involved in ubiquitin-mediated proteolysis, pyrimidine metabolism, homologous recombination and purine metabolism pathways were clearly downregulated by as little as 1 nM in Abdsc, but even higher concentrations of Dex had no effect in Om. The cell cycle pathway was also highly downregulated in Abdsc by as little as 1 nM Dex, and Om responded only to 1000 nM Dex. Together these data suggest that culture with even very low concentrations of Dex decreases cellular stress in Abdsc, but much higher concentrations are required in Om.

**Fig 1:**
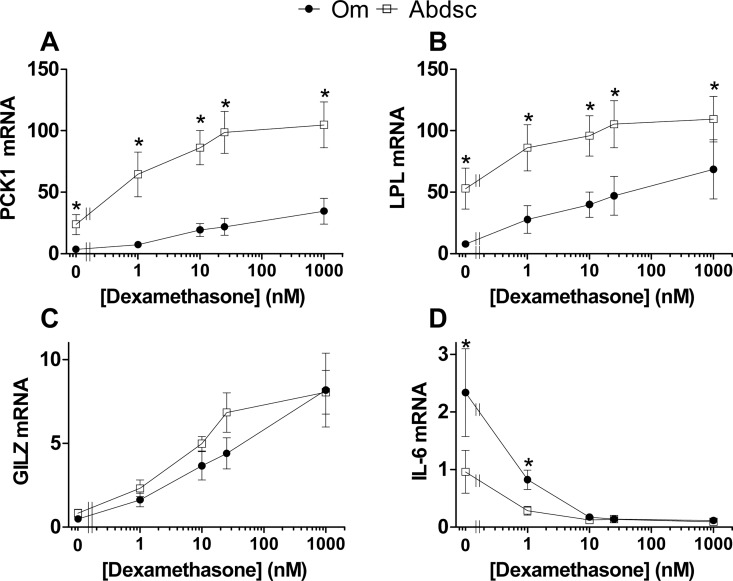
qPCR verification of concentration- and depot-dependent effects of glucocorticoids on selected, known glucocorticoid target genes. (A) *PCK1*, (B) *LPL*, (C) *GILZ*, and (D) *IL-6*. Data are mean ± SEM, n = 5–7 independent subjects. The X-axis is a log scale. Significant depot differences at each [Dex] are indicated by an asterisk (*, p < 0.05, paired t-test of log transformed values). Repeated measures ANOVA verified a significant Dex effect in both depots for each gene (Dex effect, p ≤ 0.002). All doses in both Om and Abdsc were significantly different from 0 nM Dex (p ≤ 0.05, Dunnett’s test).

*qPCR verification of depot-dependent responses to Dex*: Results for *PCK1* and *LPL*, two key enzymes that regulate TAG storage in adipocytes, were verified by qPCR (using non-pooled samples from 5–7 subjects, Fig 1). Similar to the microarray results, baseline levels of *PCK1* and *LPL* mRNA were higher in Abdsc. In addition, the sensitivity to 1 nM Dex and the magnitude of the response was higher in Abdsc for *PCK1*, but similar in the two depots for *LPL*. These results suggest that Dex, when added together with a relatively low concentration of insulin, leads to the upregulation of PPARγ signaling and therefore to a coordinated increase in the expression of key genes that regulate de novo lipogenesis and FA activation. The differential sensitivity of *PCK1* in Om vs. Abdsc contrasted with the similar dose-dependent increase in *GILZ* mRNA, a known direct target of GCs via GR. Also shown in Fig 1, as expected [14, 24], there was higher expression of *IL-6* in Om at baseline. Despite a larger absolute decrease in its expression in Om [24], sensitivity to submaximal Dex, as a % of the maximal response at each concentration, was not significantly different between the two depots (not shown), reinforcing the concept of pathway dependent differences in sensitivity and responsiveness to Dex of both metabolic and inflammatory signaling.

### Cluster analysis revealed interactions of Depot and Dex concentration on adipose tissue gene expression

**One way clustering:** To identify patterns of depot-dependent Dex effects among those transcripts that exhibited a Depot*[Dex] interaction (FDRq < 0.05) in the LME, we performed cluster analysis. Depot differences in the direction, sensitivity and magnitude of response within each cluster has the potential to suggest gene networks that are regulated in a coordinated fashion and co-factors that target them. This unbiased analysis also has the potential to identify previously unrecognized roles for GC-regulated pathways that may influence GC-mediated depot-differences in adipose tissue function.

**Fig 2:**
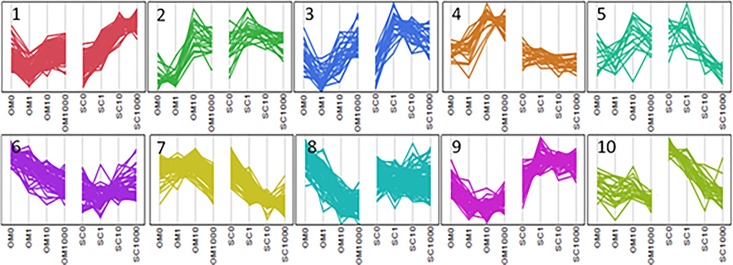
Parallel plot illustrating the cluster analysis of genes that exhibited a Depot*[Dex] interaction. 460 genes that showed a significant interaction of Depot and [Dex], and expression values above a threshold of 20 for at least one Dex concentration in one depot were included in an unsupervised hierarchical cluster analysis (JMP 10 software), as described in Methods. The analysis with 10 clusters is shown.

Using one way clustering of expression values scaled across both depots, ten clusters were identified; each was characterized by combinations of depot differences in baseline values and/or the magnitude and direction of concentration dependence of the Dex effect. Fig 2 shows a parallel plot that illustrates these patterns. Complete gene lists for the cluster analysis are given in S3 Table. Table 2 highlights depot differences in Dex effects for selected genes of interest from the viewpoint of adipose tissue metabolism, remodeling, inflammation and adipogenesis. These were selected based on 1) at least a ~2-fold response in one depot with a clearly lower in magnitude, non-existent, or opposite to the direction of the response in the other depot, or 2) depot differences in sensitivity to a submaximal concentration of Dex (1 nM) in one depot but not the other. In addition, this analysis highlighted transcripts that were regulated by Dex, but have not previously studied in the context of this tissue.

**Table 2:** Transcripts of interest from the cluster analysis of significant Depot*[Dex] interactions (Fig 2).

CLUSTER	PATTERN	GENES OF INTEREST	B IOLOGICAL PROCESS IMPLICATED
**1**	Similar baseline; Dex upregulated, greater sensitivity and response in Abdsc	*CYP4B1*, *CYP4F22*, *CYP4X1*, *CYP4Z1*	FA, steroid, lipid, xenobiotic metabolism [25]
**2**	Lower baseline in Om; Dex upregulated, lower sensitivity and greater response in Om	*CD10 (MME)*	Adipose stem cell marker [26]
		*ENPP2 (autotaxin)*	Anti-adipogenic [27]
**3**	Similar baseline; Dex upregulated, greater sensitivity in Abdsc	*LEP*	Adipokine
**4**	Similar baseline; Dex upregulated in Om, no response in Abdsc	*MUC16*	ECM, PM [28]
		*MMRN1*	PM adhesion/ coagulation [29]
		*ITLN1*	Inflammation/adipokine [30]
		*ACSM3*, *AGPAT9*	TAG synthesis [31]
**5**	Similar or higher baseline in Abdsc; Dex upregulated in Om, downregulated in Abdsc	*FADS1*	Delta 5 fatty acid desaturation
		*GPC4*	Proadipogenic; ↑insulin signaling; ECM, PM [32]
**6**	Higher baseline in Om; Dex downregulated in Om, no effects in Abdsc	*PI15*	ECM, peptidase inhibitor
		*TNFRSF21*, *LEPR*	ECM, cytokine-related
		*DDIT4*	Inhibits TORC1 signaling [33]
		*THBS2*	ECM-receptor interaction, focal adhesion; potential role in adipogenesis [34]
**7**	Similar baseline; Dex downregulated, greater sensitivity and response in Abdsc	*LTBP1*	ECM, inhibits TGFβ signaling
		*BGN*	↑ in obesity (BGN), adipose inflammation [35]
		*THBS1*	Regulates adipose expansion [36]
**8**	Higher baseline in Om; Dex downregulated in Om, lower or little response in Absc	*WNT4FLT1*	Pro-adipogenic [37]PM, pro-angiogenic; improved insulin action [38]
		*GPR116*	Cell adhesion, promotes insulin sensitivity [39]
		*INHBB*	Decrease lipolysis [40]
		*LOXL2*	Fibrosis [41]
**9**	Similar baseline; 1 nM Dex slightly downregulated in Om, upregulated in Abdsc	*NRN1*	Expressed in human adipose progenitors [26]
		*HOXC8*, *HOXC9*	Developmental/higher in Abdsc (fresh tissue) [42]
		*DKK2*	Inhibits WNT signaling (proadipogenic)
**10**	Higher baseline in Abdsc; Dex downregulated in Abdsc, lower response in Om	*GREM2*	Stimulates Wnt signaling (anti-adipogenic) [43]
		*CCL13*	Inflammation/increased in obesity[44]
		*DDIT1L*	Inhibits cell growth, TOR signaling pathway [45]

Transcripts were selected for inclusion in this Table if they exhibited a consistent ~2-fold change in one depot and the literature suggests that they may play a role in mediating depot differences in fat accumulation or depot-dependent function. Potential biological pathways/processes that may be modulated by each gene product were based on our review of the literature (Pubmed searches on the gene name and the search terms “adipose OR adipocyte”) and/or information in www.genecards.org. The complete lists of genes are in S3 Table. Plasma membrane (PM) or ECM localization of gene products is noted.

**Two-way clustering:** Two-way clustering (Depot and [Dex]) indicated that overall, values for both Om control and Om Dex 1 nM clustered with Abdsc control (0 Dex). This finding indicates that Om 1 nM values were more similar to Om control (0 Dex), but that Abdsc cultured with 1 nM Dex was different from Abdsc control and clustered with Abdsc 10 and 1000 nM and is consistent with the conclusion that overall Om is less sensitive to a low concentration of Dex. The two-way clustered dendrogram is shown in S1 Fig.

#### qPCR verification of transcripts showing depot-dependent response to Dex in organ culture

**Fig 3:**
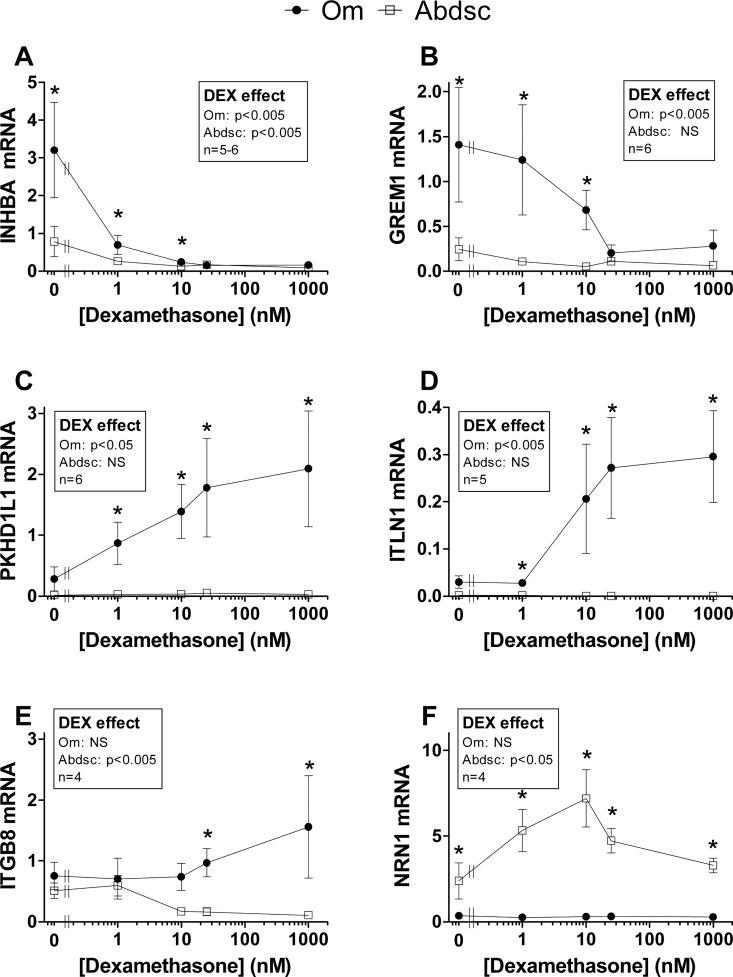
qPCR verification of selected depot-dependent Dex effects. Transcripts for verification were selected for biological interest, large depot differences in the baseline values and/or the magnitude of the Dex effects: (A) *INHBA*, (B) *GREM1*, (C) *PKHD1L1*, (D) *ITLN1*, (E) *ITGB8*, and (F) *NRN1*. Depot differences are indicated by asterisks (*p < 0.05, paired t-test at each Dex concentration). Within depot, Dex effects were tested by repeated measures ANOVA on log-transformed data (p values indicated in the box on each graph). Post-hoc comparisons of values at each Dex concentration compared to baseline (0 Dex) were carried out by Dunnett’s tests. Within Abdsc, Dex effects were significant for *INHBA* at Dex concentrations of 10 nM or higher, *ITGB8* at 25 and 1000 nM and *NRN1* at 1, 10, and 25 nM. Within Om, Dex effects were significant for *PKHD1L1* at Dex concentrations of 10 nM and higher and *ITLN1* at 25 and 1000 nM. Because of missing values for Om for *INHBA*, only paired t-tests were used to test the effect of each Dex concentration vs. baseline [p = 0.051 at 1 nM (n = 6), p<0.01 at 10 nM (n = 5), 25 and 1000 nM (n = 6)].

Using individual samples from 5–7 subjects, we verified (with qPCR) depot differences in the Dex regulation of two genes that were much more highly expressed at baseline in Om and remained higher despite suppression by Dex (*INHBA* and *GREM1*, Fig 3A and 3B), and two genes that were expressed at very low levels in Abdsc and increased by Dex only in Om (*PKHD1L1* and *ITLN1*, Fig 3C and 3D). Additionally, we confirmed the clear interaction of [Dex] and Depot for *ITGB8* (Fig 3E). This gene was suppressed by Dex only in Abdsc, while it tended to increase in response to increasing Dex in Om, creating a depot difference at the higher Dex concentrations. We also verified that *NRN1* mRNA levels were very low in Om and increased by Dex only in Abdsc (Fig 3F).

### Transcripts that showed depot differences in response to Dex also exhibit depot-differences in fresh adipose tissue

**Fig 4:**
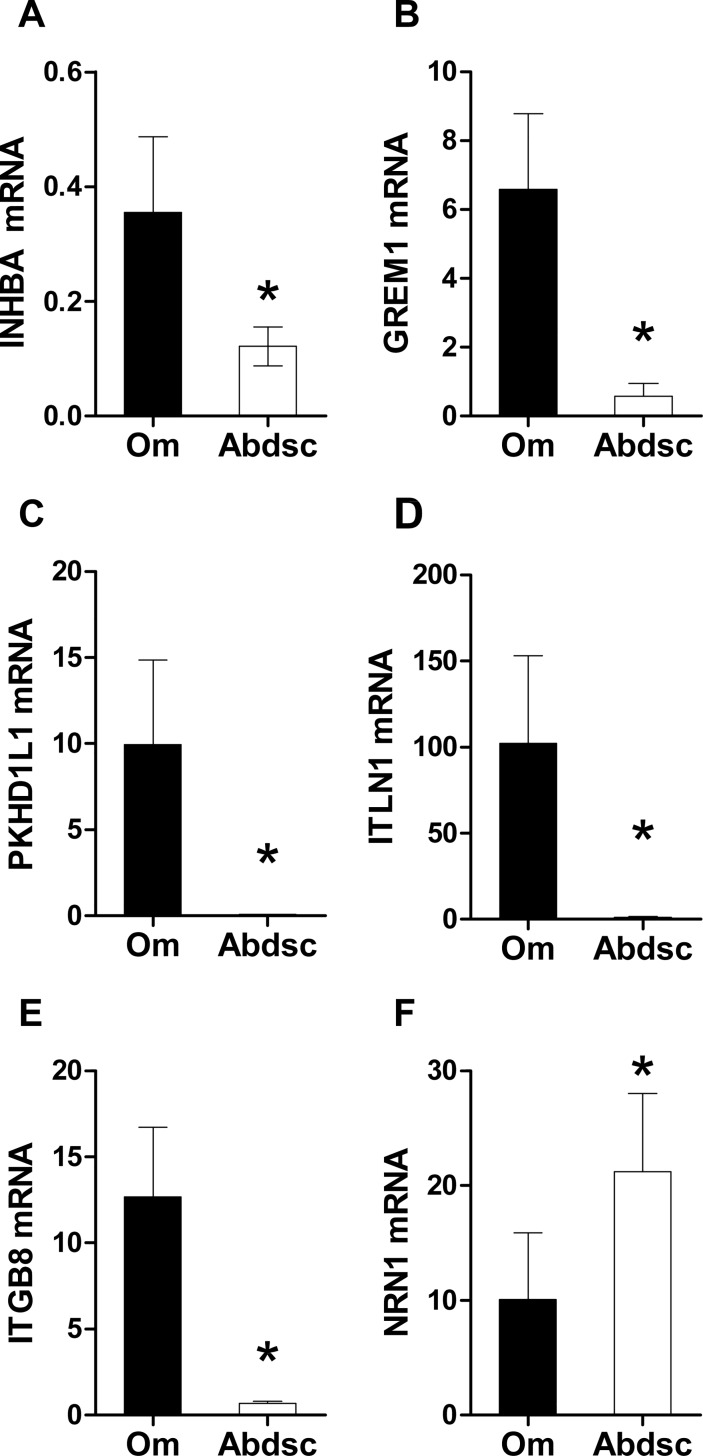
Depot differences in flash frozen samples of Om and Abdsc reflect patterns observed in tissues cultured with Dex. (A) *INHBA*, (B) *GREM1*, (C) *PKHD1L1*, (D) *ITLN1*, (E) *ITGB8*, and (F) *NRN1*. *p < 0.05, depot difference (paired t-tests of log transformed values, n = 6). Data presented as mean ± SEM.

To determine if our results in tissues cultured with Dex for 7 days ex vivo were relevant to the in vivo, we examined expression levels in tissues that were snap frozen immediately after excision. We reasoned that genes that were more highly expressed in one depot at baseline (*INHBA*, *GREM1*, and *NRN1*), and those for which a clear depot-difference was induced by culture with Dex (*ITLN1*, *PKHD1L1*, *ITGB8*, and *NRN1*) would also exhibit depot-difference in fresh adipose tissue. Fig 4 shows that genes that showed large differences after 7d of culture with Dex, *INHBA*, *GREM1*, *PKHDL1*, *ITLN1*, and *ITGB8*, were also higher in snap frozen Om, while *NRN1*, which was increased by Dex only in Abdsc, was substantially higher in fresh Abdsc tissue.

## Discussion

Although there are some developmental similarities between rodent and human visceral depots, there are also notable differences [46]. Thus, studies of mechanisms that lead to depot differences in human adipose tissue biology are especially important. The analyses presented here clearly demonstrate the depot-dependence of GC action on gene expression in human visceral (Om) compared to Abdsc adipose tissues. Using organ culture as a model system to define the long-term effects of Dex on the transcriptome of human Om and Abdsc adipose tissue, these analyses suggest potential novel molecular mechanisms underlying a broad range of well-established differences between the two depots, and point to their differential regulation by GCs. These depot differences include the higher inflammatory profile in Om adipose tissue, the lower adipogenic potential of Om preadipocytes, and the preferential accumulation of visceral fat with hypercortisolemia in vivo.

The current dataset and pathway (GSEA) analyses confirm and expand our prior observations that many metabolic (e.g. amino acid catabolism, tricarboxylic acid cycle, lipogenesis) and immune-related pathways are regulated similarly by Dex in both depots. More importantly, with the mixed model statistical approach and cluster analysis of dose-dependent changes in the adipose tissue transcriptome, we were able to identify depot-dependent differences in genes and pathways that vary in sensitivity to low Dex concentrations and others that exhibit depot differences in the magnitude or direction of the Dex effects. We verified with qPCR that selected transcripts showing depot-dependent responses to Dex in vitro (*INHBA*, *GREM1*, *ITGB8*, *ITLN1*, *PKHD1L1*, *NRN1*) also showed substantial depot differences in fresh tissue, supporting the physiological relevance of the organ culture approach and the important role of GCs in driving depot differences in adipose tissue function. These results were easily detected with a small sample size of almost exclusively female obese subjects, indicating the differences are robust and consistent.

*Dex promotion of transcripts which may modulate adipogenesis and expansion capacity in Om vs*. *Abdsc*: Excess GCs lead to the expansion of central, especially visceral adipose tissues, but the mechanisms remain poorly understood. Available data indicate that increases in both adipocyte number and size contribute to this adipose expansion [47, 48], and that a limitation on hyperplastic expansion is associated with excess hypertrophy, which in turn is associated with adipose tissue inflammation as well as adipocyte and systemic metabolic dysfunction. Our data suggest that multiple pathways that regulate adipogenesis and fat accumulation, including insulin signaling and TAG synthesis, are depot-dependently regulated by Dex in a coordinated fashion to favor the hypertrophic expansion of Om.

*Adipogenesis*: Transcript levels of autotaxin/*ENPP2* mRNA (cluster 2), which inhibits adipogenesis via increased production of lysophosphatidic acid [27], was increased by Dex in Om but unaffected in Abdsc. Additionally, the DKK2 transcripts (cluster 9), a factor which inhibits canonical anti-adipogenic Wnt signaling, were expressed at very low levels in Om and were unaffected by Dex, while Dex increased *DKK2* expression in Abdsc, potentially promoting hyperplasia in this depot. Although Dex downregulated the mRNA expression of key ligands that activate TGFβ receptor signaling (*INHBA* and *TGFβ*) and thereby inhibit adipogenesis [49, 50], they remained higher in Om compared to Abdsc adipose tissue cultured with Dex, especially at submaximal concentrations. Thus, if translated into a functional effects, these Dex-induced alterations in gene expression may mediate depot-dependent changes in hyperplastic expansion.

Additional secreted factors that may regulate TGF*β* signaling and enhance adipogenesis include, *ITGB8* [51], *LTBP1* [52] and *BGN* [53, 54] (Cluster 7). *BGN* also regulates WNT signaling [55] and adiponectin production [56]. Dex decreased *LTBP1 and BGN* levels in Abdsc, so that after Dex treatment, their levels were over 2-fold lower than in Om. However, TGF*β* expression is higher in Om, so its free concentration may still be higher in this depot. The importance of Dex in the modulation of TGF*β* activity in adipose tissues by these abundant ECM proteoglycans merits further study.

*Dex promotion of omental fat accumulation*: Our results highlight several pathways by which Dex could promote the preferential fat storage in visceral depots. Dex enhanced the expression of multiple transcripts that may promote TAG synthesis in both depots. Dex increased the expression of *GPC4* mRNA, which encodes a secreted factor (adipokine), only in Om (cluster 5). Based on studies in mouse adipocytes, *GPC4* is thought to promote insulin sensitivity, and clinical studies suggest that higher expression of *GPC4* is associated with higher waist-to-hip ratio and visceral to Abdsc fat ratio in men [32]. *INHBB* (cluster 8), which is increased by adipocyte differentiation and expressed by mature adipocytes and decreases lipolysis [40], was decreased by Dex only in Om, resulting in higher levels in Abdsc than Om (confirmed with qPCR, data not shown). *INHBB* expression was also higher in fresh Abdsc than Om adipose tissue (2.7 ± 0.9 vs. 1.3 ± 0.6, relative expression by qPCR, p<0.05, n = 6). At the same time, transcript levels of genes likely involved in FA activation (*ACSM3*) and esterification (*AGPAT9*) [31] were increased by Dex only in Om (Cluster 4), which could balance the higher lipolysis. These changes are consistent with findings in mice which document that GCs promotes high TAG turnover [57], and intriguingly suggest a viscerally specific effect that merits further investigation as a mechanism to link hypercortisolemia to visceral fat accumulation and metabolic dysregulation. Finally, Dex treatment led to a larger fold increase (1.6 fold in Om vs. 3.4 fold in Abdsc, p<0.05) in expression of PPARGC1A, a transcriptional co-activator that plays a major role in increasing genes that regulate oxidative metabolism and thereby the metabolic health of adipocytes.

*Response to stress/mTOR signaling*: Genes induced by cellular stress such as *DDIT4L* (*REDD2*), which inhibits mTORC1 [45], was more highly expressed at baseline in Abdsc and decreased by Dex in both depots. In addition, *DDIT4* (*REDD1*) inhibits mTORC1 and is a negative regulator of insulin signaling [33], so its decrease by Dex in only Om, is consistent with an enhancement of insulin action and lipogenesis in adipocytes. Overall these results suggest that Dex restrains the cellular stress in adipose tissue and thereby improves insulin action.

*Additional transcripts that encode factors that modulate lipid metabolism especially in Abdsc*: A number of transcripts that regulate lipid metabolism were more potently induced by Dex in Abdsc. Three members of the Cytochrome P450, Family 4 (*CYP4B1*, *CYP4F22* and *CYP4X1*) clustered together (Cluster 1). For example, *CYP4B1* was expressed at a very low level under baseline conditions in Om and increased by Dex by 8-fold compared to 40-fold in Abdsc. These CYP4 genes have ill-defined roles in FA, steroid, lipid and xenobiotic metabolism [25].

In addition to the similar Dex stimulation of genes encoding transcripts involved in the synthesis of very long chain fatty acids and delta 9 desaturation in Om and Abdsc, Dex differentially regulated FADS1 (delta 5 desaturase), which was increased by 50% in Om but decreased by 10% in Abdsc, and *FADS2* (delta 6 desaturase), which did not change in Om and was decreased by Dex (30%) in Abdsc. Depot differences in the effect of Dex on these genes of FA metabolism have potential for regulating tissue FA composition, biophysical properties of membranes, and production of functionally important lipid mediators.

*Inflammation and immunity*: Culture with Dex led to a more robust increase in Abdsc than Om of *CD300LG* which is implicated in T-cell recruitment [58] and *P2RY14/GPR105* (Cluster 1) which modulates macrophage recruitment in diet-induced obesity [58–60]. Cytokine-related genes (*TNFRSF21*, *IL1RAP*, *TSLP*, and *LEPR*) also followed this pattern, which is somewhat surprising given reports of higher inflammation in Om and Abdsc. *STEAP4/STAMP2* mRNA, which decreases adipose inflammation and enhances adipocyte insulin sensitivity [61, 62], and is also important for promoting fat accumulation, was more responsive to Dex stimulation in Abdsc than Om (Cluster 1). The chemokine *CCL13* (*MCP-4*) was more highly expressed in Abdsc at baseline and suppressed by Dex only in that depot [63]. Taken together, there data suggest the depot differences in mechanisms by which GCs regulate inflammatory pathways and immune cell recruitment.

*Dex induced a cluster of genes only in Om*: Of the 20 genes in Cluster 4, 7 had similar levels of baseline expression and showed a clear dose-dependent increase in response to increasing concentrations of Dex in Om, but had very low expression and no response to Dex in Abdsc (*MUC16*, *ART4*, *MMRN1*, and *MUM1L1*). *ITLN1* (omentin), a gene which is documented to be specific for visceral adipose tissue [30], also displayed this pattern (confirmed by qPCR, Figs 3 and 4). Genes that were much more highly expressed in Om included mesothelin, a marker of mesothelial cells, as previously observed by others [64]. *MUC16*, *MMRN1*, *ITLN1*, and *IL-18* were also upregulated by Dex in Om and are known to be more abundantly expressed in fresh-frozen Om than Abdsc samples ([30, 65] and our unpublished data). Mucin 16 is a cell surface receptor involved in cell adhesion whose extracellular domain is secreted and is known to bind to mesothelin, a cell surface protein on mesothelial cells [28]. Further studies of the importance of mesothelial cells in the biology of human visceral adipose tissue are warranted, especially in view of a recent lineage tracing study which indicates that adipocytes in visceral depots derive from a mesothelial lineage in mice [66].

*Cell adhesion and/or migration*: A number of genes in Cluster 6 were higher at baseline and decreased by Dex in Om, but were affected little by Dex in Abdsc. 11 genes in this list were functionally classified as being involved in Cell Adhesion and/or Migration (DAVID analysis) [67, 68] including *GPR56*, *AMIGO2*, *CDH2*, *CLSTN1*, and *THBS2*. In addition, *GPR116*, which suppresses cell migration was higher in Om at baseline and yet more robustly downregulated by low Dex concentrations. Overall, these data indicate that Dex has depot-dependent effects on cell adhesion and migration pathways and thus potentially on immune function and other processes that affect inflammation and remodeling. Combined with the higher baseline yet similar relative magnitude of the potent suppressive effects of Dex in both Om and Abdsc adipose tissues, these additional players may have differential effects on the fine-tuning control of inflammation within each depot.

*Additional transcripts regulated in a Depot-specific and Dex-dependent manner*: *PI15* (peptidase inhibitor 15, also known as protease inhibitor 15 and *CRISP8*) is a trypsin inhibitor that was expressed at fairly high levels and dose-dependently and markedly decreased by Dex (by ~10-fold) in Om, while it was very low at baseline and decreased only ~2-fold in Abdsc. Little is known about this gene, and nothing about its function in adipose tissue, but it was identified as a gene with a glucocorticoid binding region by ChIP-seq in 3T3-L1 adipocytes [57]. Cluster 9 included 50 genes that were higher in Abdsc than Om at baseline. Especially dramatic was *NRN1* (Neuritin 1). Its expression was ~6–7 fold lower and unchanged by Dex in Om, while in Abdsc it was dose-dependently increased (2-fold), resulting in a ~12 fold depot difference at maximal Dex (as verified by qPCR, Fig 3F). Very little is known about this gene other than it is likely GPI-anchored to the plasma membrane and expressed in the central nervous system where it promotes neurite outgrowth [69] and is induced by hypoxia in tumors [70]. *PKHD1L1* was increased by Dex only in Om to fairly high levels (Fig 3), but little is known of its function in any context.

A limitation of this study is that the number of subjects studied in the initial microarray was small (n = 3 representing a total of 5 subjects), all were severely obese, and 6/7 were women. However, results were robust and key findings were easily confirmed with n = 5–7 by qPCR. These studies were not powered to detect differences in sensitivity or responsiveness to Dex effects between lean vs. obese or female vs. males. Further studies are needed to verify additional genes of interest and assess how depot-dependent Dex action varies as a function of level of adiposity, age, sex, fat distribution and metabolic status. An additional limitation of our studies was that we only tested Dex, and not cortisol, the physiological ligand, which acts on both MR and GR. We chose Dex, a specific type II GR agonist, to avoid potential confounds of depot differences in cortisone/cortisol activation [71]. In one of the subjects, we performed microarray study comparing the effects of cortisol (200 nM) and Dex in both Om and Abdsc, found that that effects are similar and in fact the correlation of the microarray data for cortisol (200 nM) and Dex (10 nM) was over r = 0.95.

It is important to note that our organ culture system includes insulin (0.7 nM), and we and others find the combination of insulin and Dex best promotes the expression of genes encoding enzymes that regulate fat metabolism and adipokines [13, 14]. A recent ChIP study in human adipose tissue cultured with Dex but without insulin reported similar results with regard to inflammation pathways, but did not observe changes in de novo lipogenic and TAG synthesis pathways [72].

In summary, this study reinforces our knowledge of the pleiotropic effects of GCs on transcripts expressed in two major human adipose tissues, and points to the need for understanding gene- and cell-dependent variations in sensitivity to GC action. The mechanistic basis for the differential sensitivity and responsiveness to GCs in each cell type is likely to occur via differences in the level of transcriptional co-activators and repressors, as well as changes in GR phosphorylation that may depend on the level of inflammation [10, 73]. We found that phosphorylation of GR at serine 226 is higher in Om vs. Abdsc adipose tissue (Lee MJ and Fried SK, unpublished observation) which may contribute to the lower sensitivity to GCs in the former. Additionally, because adipose tissue includes multiple cell types, it is tempting to speculate that cell-specific actions of GCs via GR contribute to depot differences in their function. These hypothesis-generating analyses emphasize the need to determine the cell types that express the secreted factors, establish which effects are cell autonomous, define the Dex-regulated components of the ECM that contribute to the microenvironment within specific visceral and sc depots, and determine their functional roles as well as which transcripts are direct vs. secondary targets of GCs/GR [12]. Future studies of the importance of GCs in modulating the pathways predicted to contribute to each depot’s unique functions and expansion capacity have clear potential for understanding of human fat distribution and its impact on metabolic health.

## Supporting Information

S1 Fig Dendrogram of 2 way cluster analysis of transcripts exhibiting a Depot*[Dex] interaction. The intensity of the red or blue color in each row represents expression values, high or low respectively, which were scaled across both depots, and each column represents the culture condition (culture with 0, 1, 10, or 1000 nM Dex is designated Om0, Om1, Om10, Om1000, Abdsc0, Abdsc1, Abdsc10, Abdsc1000) as described in Methods.
(TIF)

S1 Table Expression values for all transcripts in the microarray. Averages of expression values (linear scale) at each Dex concentration are given (average values of n = 3 subjects). FDRq for LME parameters (Depot, [Dex], and Depot*[Dex] interaction) are given for each gene.
(XLSX)

S2 Table Lists of pathways identified by GSEA as up- or down-regulated by each concentration of Dex in both depots (S2A and S2B, respectively), or only in 1 depot by Dex (S2C). Gene lists for each depots were preranked by fold change vs. 0 nM Dex and analyzed by GSEA, as described in Methods. FDRq values < 0.05 were considered statistically significant.
(XLSX)

S3 Table Lists of the 460 transcripts used for cluster analysis in Fig 3. Genes exhibiting significant Depot × [Dex] interaction (p < 0.05) were used for this cluster analysis. Cluster and sub-cluster numbers as well as LME results and expression values are given for each gene [Data are the mean of the expression values (linearized scale) for the 3 paired samples of Om and Abdsc from the microarray analysis].
(XLSX)

## References

[1] Tchernof A, Despres JP. Pathophysiology of human visceral obesity: an update. Physiol Rev. 2013;93(1):359–404. 10.1152/physrev.00033.2011 23303913

[2] Geer EB, Shen W, Strohmayer E, Post KD, Freda PU. Body composition and cardiovascular risk markers after remission of Cushing's disease: a prospective study using whole-body MRI. J Clin Endocrinol Metab. 2012;97(5):1702–11. 10.1210/jc.2011-3123 22419708 PMC3339890

[3] Geer EB, Shen W, Gallagher D, Punyanitya M, Looker HC, Post KD, et al MRI assessment of lean and adipose tissue distribution in female patients with Cushing's disease. Clin Endocrinol (Oxf). 2010;73(4):469–75. PubMed Central PMCID: PMCPMC2962672. 20550536 10.1111/j.1365-2265.2010.03829.x PMC2962672

[4] Wajchenberg BL, Bosco A, Marone MM, Levin S, Rocha M, Lerario AC, et al Estimation of body fat and lean tissue distribution by dual energy X-ray absorptiometry and abdominal body fat evaluation by computed tomography in Cushing's disease. J Clin Endocrinol Metab. 1995;80(9):2791–4. 10.1210/jcem.80.9.7673425 7673425

[5] Masuzaki H, Paterson J, Shinyama H, Morton NM, Mullins JJ, Seckl JR, et al A transgenic model of visceral obesity and the metabolic syndrome. Science. 2001;294(5549):2166–70. 10.1126/science.1066285 11739957

[6] Drolet R, Richard C, Sniderman AD, Mailloux J, Fortier M, Huot C, et al Hypertrophy and hyperplasia of abdominal adipose tissues in women. Int J Obes (Lond). 2008;32(2):283–91. 17726433 10.1038/sj.ijo.0803708

[7] Tchernof A, Belanger C, Morisset AS, Richard C, Mailloux J, Laberge P, et al Regional differences in adipose tissue metabolism in women: minor effect of obesity and body fat distribution. Diabetes. 2006;55(5):1353–60. 16644692 10.2337/db05-1439

[8] Patel R, Williams-Dautovich J, Cummins CL. Minireview: new molecular mediators of glucocorticoid receptor activity in metabolic tissues. Mol Endocrinol. 2014;28(7):999–1011. 10.1210/me.2014-1062 24766141 PMC5414825

[9] Chodankar R, Wu DY, Schiller BJ, Yamamoto KR, Stallcup MR. Hic-5 is a transcription coregulator that acts before and/or after glucocorticoid receptor genome occupancy in a gene-selective manner. Proc Natl Acad Sci U S A. 2014;111(11):4007–12. 10.1073/pnas.1400522111 24591583 PMC3964041

[10] Vandevyver S, Dejager L, Libert C. Comprehensive overview of the structure and regulation of the glucocorticoid receptor. Endocr Rev. 2014;35(4):671–93. 10.1210/er.2014-1010 24937701

[11] Lee MJ, Wu Y, Fried SK. Adipose tissue heterogeneity: implication of depot differences in adipose tissue for obesity complications. Mol Aspects Med. 2013;34(1):1–11. 10.1016/j.mam.2012.10.001 23068073 PMC3549425

[12] Grandl G, Muller S, Moest H, Moser C, Wollscheid B, Wolfrum C. Depot specific differences in the adipogenic potential of precursors are mediated by collagenous extracellular matrix and Flotillin 2 dependent signaling. Mol Metab. 2016;5(10):937–47. 10.1016/j.molmet.2016.07.008 27689006 PMC5034610

[13] Fried SK, Russell CD, Grauso NL, Brolin RE. Lipoprotein lipase regulation by insulin and glucocorticoid in subcutaneous and omental adipose tissues of obese women and men. J Clin Invest. 1993;92(5):2191–8. 10.1172/JCI116821 8227334 PMC288398

[14] Lee MJ, Gong DW, Burkey BF, Fried SK. Pathways regulated by glucocorticoids in omental and subcutaneous human adipose tissues: a microarray study. Am J Physiol Endocrinol Metab. 2011;300(3):E571–E80. 10.1152/ajpendo.00231.2010 21189358 PMC3279304

[15] Lee MJ, Wang Y, Ricci MR, Sullivan S, Russell CD, Fried SK. Acute and chronic regulation of leptin synthesis, storage, and secretion by insulin and dexamethasone in human adipose tissue. Am J Physiol Endocrinol Metab. 2007;292(3):E858–E64. 10.1152/ajpendo.00439.2006 17122089

[16] Vockley CM, D'Ippolito AM, McDowell IC, Majoros WH, Safi A, Song L, et al Direct GR Binding Sites Potentiate Clusters of TF Binding across the Human Genome. Cell. 2016;166(5):1269–81 e19. 10.1016/j.cell.2016.07.049 27565349 PMC5046229

[17] Roqueta-Rivera M, Esquejo RM, Phelan PE, Sandor K, Daniel B, Foufelle F, et al SETDB2 Links Glucocorticoid to Lipid Metabolism through Insig2a Regulation. Cell Metab. 2016;24(3):474–84. PubMed Central PMCID: PMCPMC5023502. 10.1016/j.cmet.2016.07.025 27568546 PMC5023502

[18] Fried SK, Moustaid-Moussa N. Culture of adipose tissue and isolated adipocytes. Methods Mol Biol. 2001;155:197–212. 10.1385/1-59259-231-7:197 11293072

[19] Mootha VK, Lindgren CM, Eriksson KF, Subramanian A, Sihag S, Lehar J, et al PGC-1alpha-responsive genes involved in oxidative phosphorylation are coordinately downregulated in human diabetes. Nat Genet. 2003;34(3):267–73. 10.1038/ng1180 12808457

[20] Subramanian A, Tamayo P, Mootha VK, Mukherjee S, Ebert BL, Gillette MA, et al Gene set enrichment analysis: a knowledge-based approach for interpreting genome-wide expression profiles. Proc Natl Acad Sci U S A. 2005;102(43):15545–50. 10.1073/pnas.0506580102 16199517 PMC1239896

[21] Nye CK, Hanson RW, Kalhan SC. Glyceroneogenesis is the dominant pathway for triglyceride glycerol synthesis in vivo in the rat. J Biol Chem. 2008;283(41):27565–74. PubMed Central PMCID: PMCPMC2562054. 10.1074/jbc.M804393200 18662986 PMC2562054

[22] Yasmeen R, Jeyakumar SM, Reichert B, Yang F, Ziouzenkova O. The contribution of vitamin A to autocrine regulation of fat depots. Biochim Biophys Acta. 2012;1821(1):190–7. PubMed Central PMCID: PMCPMC3196743. 10.1016/j.bbalip.2011.06.004 21704731 PMC3196743

[23] Catalan V, Gomez-Ambrosi J, Rodriguez A, Ramirez B, Rotellar F, Valenti V, et al Increased tenascin C and Toll-like receptor 4 levels in visceral adipose tissue as a link between inflammation and extracellular matrix remodeling in obesity. J Clin Endocrinol Metab. 2012;97(10):E1880–9. PubMed Central PMCID: PMCPMC3462948. 10.1210/jc.2012-1670 22851489 PMC3462948

[24] Fried SK, Bunkin DA, Greenberg AS. Omental and subcutaneous adipose tissues of obese subjects release interleukin-6: depot difference and regulation by glucocorticoid. J Clin Endocrinol Metab. 1998;83(3):847–50. 10.1210/jcem.83.3.4660 9506738

[25] Hsu MH, Savas U, Griffin KJ, Johnson EF. Human cytochrome p450 family 4 enzymes: function, genetic variation and regulation. Drug Metab Rev. 2007;39(2–3):515–38. 10.1080/03602530701468573 17786636

[26] Tews D, Schwar V, Scheithauer M, Weber T, Fromme T, Klingenspor M, et al Comparative gene array analysis of progenitor cells from human paired deep neck and subcutaneous adipose tissue. Mol Cell Endocrinol. 2014;395(1–2):41–50. 10.1016/j.mce.2014.07.011 25102227

[27] Nishimura S, Nagasaki M, Okudaira S, Aoki J, Ohmori T, Ohkawa R, et al ENPP2 contributes to adipose tissue expansion and insulin resistance in diet-induced obesity. Diabetes. 2014;63(12):4154–64. 10.2337/db13-1694 24969110

[28] Rump A, Morikawa Y, Tanaka M, Minami S, Umesaki N, Takeuchi M, et al Binding of ovarian cancer antigen CA125/MUC16 to mesothelin mediates cell adhesion. J Biol Chem. 2004;279(10):9190–8. 10.1074/jbc.M312372200 14676194

[29] Jeimy SB, Fuller N, Tasneem S, Segers K, Stafford AR, Weitz JI, et al Multimerin 1 binds factor V and activated factor V with high affinity and inhibits thrombin generation. Thromb Haemost. 2008;100(6):1058–67. 19132231

[30] Yang RZ, Lee MJ, Hu H, Pray J, Wu HB, Hansen BC, et al Identification of omentin as a novel depot-specific adipokine in human adipose tissue: possible role in modulating insulin action. Am J Physiol Endocrinol Metab. 2006;290(6):E1253–61. 10.1152/ajpendo.00572.2004 16531507

[31] Shan D, Li JL, Wu L, Li D, Hurov J, Tobin JF, et al GPAT3 and GPAT4 are regulated by insulin-stimulated phosphorylation and play distinct roles in adipogenesis. J Lipid Res. 2010;51(7):1971–81. PubMed Central PMCID: PMCPMC2882735. 10.1194/jlr.M006304 20181984 PMC2882735

[32] Yoo HJ, Hwang SY, Cho GJ, Hong HC, Choi HY, Hwang TG, et al Association of glypican-4 with body fat distribution, insulin resistance, and nonalcoholic fatty liver disease. J Clin Endocrinol Metab. 2013;98(7):2897–901. 10.1210/jc.2012-4297 23633195

[33] Regazzetti C, Dumas K, Le Marchand-Brustel Y, Peraldi P, Tanti JF, Giorgetti-Peraldi S. Regulated in development and DNA damage responses -1 (REDD1) protein contributes to insulin signaling pathway in adipocytes. PLoS One. 2012;7(12):e52154 PubMed Central PMCID: PMCPMC3525563. 10.1371/journal.pone.0052154 23272222 PMC3525563

[34] Shitaye HS, Terkhorn SP, Combs JA, Hankenson KD. Thrombospondin-2 is an endogenous adipocyte inhibitor. Matrix Biol. 2010;29(6):549–56. PubMed Central PMCID: PMCPMC2939302. 10.1016/j.matbio.2010.05.006 20561899 PMC2939302

[35] Matsuo Y, Tanaka M, Yamakage H, Sasaki Y, Muranaka K, Hata H, et al Thrombospondin 1 as a novel biological marker of obesity and metabolic syndrome. Metabolism. 2015;64(11):1490–9. PubMed Central PMCID: PMCPMC4936918. 10.1016/j.metabol.2015.07.016 26298466 PMC4936918

[36] Kong P, Gonzalez-Quesada C, Li N, Cavalera M, Lee DW, Frangogiannis NG. Thrombospondin-1 regulates adiposity and metabolic dysfunction in diet-induced obesity enhancing adipose inflammation and stimulating adipocyte proliferation. Am J Physiol Endocrinol Metab. 2013;305(3):E439–50. PubMed Central PMCID: PMCPMC3742854. 10.1152/ajpendo.00006.2013 23757408 PMC3742854

[37] Nishizuka M, Koyanagi A, Osada S, Imagawa M. Wnt4 and Wnt5a promote adipocyte differentiation. FEBS Lett. 2008;582(21–22):3201–5. 10.1016/j.febslet.2008.08.011 18708054

[38] Robciuc MR, Kivela R, Williams IM, de Boer JF, van Dijk TH, Elamaa H, et al VEGFB/VEGFR1-Induced Expansion of Adipose Vasculature Counteracts Obesity and Related Metabolic Complications. Cell Metab. 2016;23(4):712–24. 10.1016/j.cmet.2016.03.004 27076080 PMC5898626

[39] Nie T, Hui X, Gao X, Li K, Lin W, Xiang X, et al Adipose tissue deletion of Gpr116 impairs insulin sensitivity through modulation of adipose function. FEBS Lett. 2012;586(20):3618–25. 10.1016/j.febslet.2012.08.006 22971422

[40] Magnusson B, Svensson PA, Carlsson LM, Sjoholm K. Activin B inhibits lipolysis in 3T3-L1 adipocytes. Biochem Biophys Res Commun. 2010;395(3):373–6. 10.1016/j.bbrc.2010.04.022 20382119

[41] Garner JA. Differential turnover of tubulin and neurofilament proteins in central nervous system neuron terminals. Brain Res. 1988;458(2):309–18. 2463048 10.1016/0006-8993(88)90473-8

[42] Yamamoto Y, Gesta S, Lee KY, Tran TT, Saadatirad P, Kahn CR. Adipose depots possess unique developmental gene signatures. Obesity (Silver Spring). 2010;18(5):872–8. PubMed Central PMCID: PMCPMC4377838. 20111017 10.1038/oby.2009.512 PMC4377838

[43] Wu Q, Tang SG, Yuan ZM. Gremlin 2 inhibits adipocyte differentiation through activation of Wnt/beta-catenin signaling. Mol Med Rep. 2015;12(4):5891–6. 10.3892/mmr.2015.4117 26239165

[44] Hashimoto I, Wada J, Hida A, Baba M, Miyatake N, Eguchi J, et al Elevated serum monocyte chemoattractant protein-4 and chronic inflammation in overweight subjects. Obesity (Silver Spring). 2006;14(5):799–811. 16855189 10.1038/oby.2006.93

[45] Corradetti MN, Inoki K, Guan KL. The stress-inducted proteins RTP801 and RTP801L are negative regulators of the mammalian target of rapamycin pathway. J Biol Chem. 2005;280(11):9769–72. 10.1074/jbc.C400557200 15632201

[46] Gesta S, Bluher M, Yamamoto Y, Norris AW, Berndt J, Kralisch S, et al Evidence for a role of developmental genes in the origin of obesity and body fat distribution. Proc Natl Acad Sci U S A. 2006;103(17):6676–81. 10.1073/pnas.0601752103 16617105 PMC1458940

[47] Lessard J, Laforest S, Pelletier M, Leboeuf M, Blackburn L, Tchernof A. Low abdominal subcutaneous preadipocyte adipogenesis is associated with visceral obesity, visceral adipocyte hypertrophy, and a dysmetabolic state. Adipocyte. 2014;3(3):197–205. PubMed Central PMCID: PMCPMC4110096. 10.4161/adip.29385 25068086 PMC4110096

[48] Arner P, Andersson DP, Thorne A, Wiren M, Hoffstedt J, Naslund E, et al Variations in the size of the major omentum are primarily determined by fat cell number. J Clin Endocrinol Metab. 2013;98(5):E897–901. 10.1210/jc.2012-4106 23543656

[49] Zaragosi LE, Wdziekonski B, Villageois P, Keophiphath M, Maumus M, Tchkonia T, et al Activin a plays a critical role in proliferation and differentiation of human adipose progenitors. Diabetes. 2010;59(10):2513–21. PubMed Central PMCID: PMCPMC3279533. 10.2337/db10-0013 20530742 PMC3279533

[50] Du B, Cawthorn WP, Su A, Doucette CR, Yao Y, Hemati N, et al The transcription factor paired-related homeobox 1 (Prrx1) inhibits adipogenesis by activating transforming growth factor-beta (TGFbeta) signaling. J Biol Chem. 2013;288(5):3036–47. PubMed Central PMCID: PMCPMC3561528. 10.1074/jbc.M112.440370 23250756 PMC3561528

[51] Aluwihare P, Mu Z, Zhao Z, Yu D, Weinreb PH, Horan GS, et al Mice that lack activity of alphavbeta6- and alphavbeta8-integrins reproduce the abnormalities of Tgfb1- and Tgfb3-null mice. J Cell Sci. 2009;122(Pt 2):227–32. PubMed Central PMCID: PMCPMC2714418. 10.1242/jcs.035246 19118215 PMC2714418

[52] Drews F, Knobel S, Moser M, Muhlack KG, Mohren S, Stoll C, et al Disruption of the latent transforming growth factor-beta binding protein-1 gene causes alteration in facial structure and influences TGF-beta bioavailability. Biochim Biophys Acta. 2008;1783(1):34–48. 10.1016/j.bbamcr.2007.08.004 17950478

[53] Tang T, Thompson JC, Wilson PG, Nelson C, Williams KJ, Tannock LR. Decreased body fat, elevated plasma transforming growth factor-beta levels, and impaired BMP4-like signaling in biglycan-deficient mice. Connect Tissue Res. 2013;54(1):5–13. 10.3109/03008207.2012.715700 22834985 PMC4557867

[54] Hara T, Yoshida E, Shinkai Y, Yamamoto C, Fujiwara Y, Kumagai Y, et al Biglycan Intensifies ALK5-Smad2/3 Signaling by TGF-beta1 and Downregulates Syndecan-4 in Cultured Vascular Endothelial Cells. J Cell Biochem. 2016. 10.1002/jcb.25721 PMC6221004 27585241

[55] Berendsen AD, Fisher LW, Kilts TM, Owens RT, Robey PG, Gutkind JS, et al Modulation of canonical Wnt signaling by the extracellular matrix component biglycan. Proc Natl Acad Sci U S A. 2011;108(41):17022–7. PubMed Central PMCID: PMCPMC3193219. 10.1073/pnas.1110629108 21969569 PMC3193219

[56] Ward MG, Ajuwon KM. Biglycan deletion alters adiponectin expression in murine adipose tissue and 3T3-L1 adipocytes. PLoS One. 2012;7(11):e50554 PubMed Central PMCID: PMCPMC3506581. 10.1371/journal.pone.0050554 23189205 PMC3506581

[57] Yu CY, Mayba O, Lee JV, Tran J, Harris C, Speed TP, et al Genome-wide analysis of glucocorticoid receptor binding regions in adipocytes reveal gene network involved in triglyceride homeostasis. PLoS One. 2010;5(12):e15188 10.1371/journal.pone.0015188 21187916 PMC3004788

[58] Umemoto E, Takeda A, Jin S, Luo Z, Nakahogi N, Hayasaka H, et al Dynamic changes in endothelial cell adhesion molecule nepmucin/CD300LG expression under physiological and pathological conditions. PLoS One. 2013;8(12):e83681 10.1371/journal.pone.0083681 24376728 PMC3871519

[59] Arase T, Uchida H, Kajitani T, Ono M, Tamaki K, Oda H, et al The UDP-glucose receptor P2RY14 triggers innate mucosal immunity in the female reproductive tract by inducing IL-8. J Immunol. 2009;182(11):7074–84. 10.4049/jimmunol.0900001 19454705

[60] Xu J, Morinaga H, Oh D, Li P, Chen A, Talukdar S, et al GPR105 ablation prevents inflammation and improves insulin sensitivity in mice with diet-induced obesity. J Immunol. 2012;189(4):1992–9. 10.4049/jimmunol.1103207 22778393 PMC3411902

[61] Wellen KE, Fucho R, Gregor MF, Furuhashi M, Morgan C, Lindstad T, et al Coordinated regulation of nutrient and inflammatory responses by STAMP2 is essential for metabolic homeostasis. Cell. 2007;129(3):537–48. 10.1016/j.cell.2007.02.049 17482547 PMC2408881

[62] Moreno-Navarrete JM, Ortega F, Serrano M, Perez-Perez R, Sabater M, Ricart W, et al Decreased STAMP2 expression in association with visceral adipose tissue dysfunction. J Clin Endocrinol Metab. 2011;96(11):E1816–E25. 10.1210/jc.2011-0310 21849520

[63] Hashimoto I, Wada J, Hida A, Baba M, Miyatake N, Eguchi J, et al Elevated serum monocyte chemoattractant protein-4 and chronic inflammation in overweight subjects. Obesity (Silver Spring). 2006;14(5):799–811. 16855189 10.1038/oby.2006.93

[64] Darimont C, Avanti O, Blancher F, Wagniere S, Mansourian R, Zbinden I, et al Contribution of mesothelial cells in the expression of inflammatory-related factors in omental adipose tissue of obese subjects. Int J Obes (Lond). 2008;32(1):112–20. 17637700 10.1038/sj.ijo.0803688

[65] Hardy OT, Perugini RA, Nicoloro SM, Gallagher-Dorval K, Puri V, Straubhaar J, et al Body mass index-independent inflammation in omental adipose tissue associated with insulin resistance in morbid obesity. Surg Obes Relat Dis. 2011;7(1):60–7. 10.1016/j.soard.2010.05.013 20678967 PMC2980798

[66] Chau YY, Bandiera R, Serrels A, Martinez-Estrada OM, Qing W, Lee M, et al Visceral and subcutaneous fat have different origins and evidence supports a mesothelial source. Nat Cell Biol. 2014;16(4):367–75. 10.1038/ncb2922 24609269 PMC4060514

[67] Huang DW, Sherman BT, Tan Q, Kir J, Liu D, Bryant D, et al DAVID Bioinformatics Resources: expanded annotation database and novel algorithms to better extract biology from large gene lists. Nucleic Acids Res. 2007;35(Web Server issue):W169–W75. 10.1093/nar/gkm415 17576678 PMC1933169

[68] Huang dW, Sherman BT, Lempicki RA. Bioinformatics enrichment tools: paths toward the comprehensive functional analysis of large gene lists. Nucleic Acids Res. 2009;37(1):1–13. 10.1093/nar/gkn923 19033363 PMC2615629

[69] Zhou S, Zhou J. Neuritin, a neurotrophic factor in nervous system physiology. Curr Med Chem. 2014;21(10):1212–9. 24350851 10.2174/0929867321666131218093327

[70] Le JS, Le MN, Cazes A, Philippe J, Le CM, Leger J, et al Characterization of the expression of the hypoxia-induced genes neuritin, TXNIP and IGFBP3 in cancer. FEBS Lett. 2006;580(14):3395–400. 10.1016/j.febslet.2006.05.011 16723126

[71] Lee MJ, Fried SK. The glucocorticoid receptor, not the mineralocorticoid receptor, plays the dominant role in adipogenesis and adipokine production in human adipocytes. Int J Obes (Lond). 2014;38(9):1228–33. PubMed Central PMCID: PMCPMC4321810. 24430397 10.1038/ijo.2014.6 PMC4321810

[72] Singh P, Brock CO, Volden PA, Hernandez K, Skor M, Kocherginsky M, et al Glucocorticoid receptor ChIP-sequencing of subcutaneous fat reveals modulation of inflammatory pathways. Obesity (Silver Spring). 2015;23(11):2286–93. PubMed Central PMCID: PMCPMC4818951. 26408078 10.1002/oby.21251 PMC4818951

[73] Chen W, Dang T, Blind RD, Wang Z, Cavasotto CN, Hittelman AB, et al Glucocorticoid receptor phosphorylation differentially affects target gene expression. Mol Endocrinol. 2008;22(8):1754–66. 10.1210/me.2007-0219 18483179 PMC2725771

