# Supplementary figures and images for: Depot Dependent Effects of Dexamethasone on Gene Expression in Human Omental and Abdominal Subcutaneous Adipose Tissues from Obese Women
Source: PLoS One. 2016 Dec 22;11(12):e0167337. doi: 10.1371/journal.pone.0167337 (PMC5179014; doi:10.1371/journal.pone.0167337)

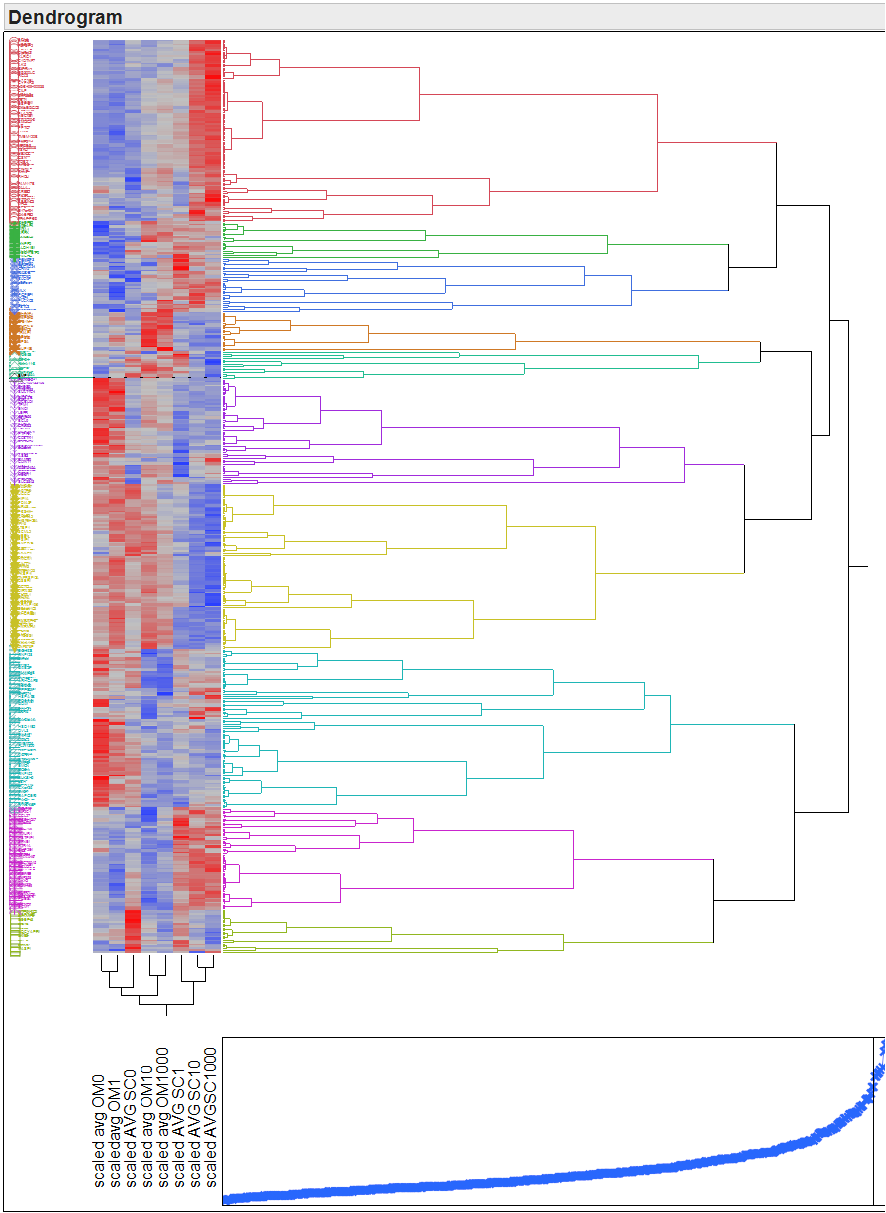

Supplement: S1 Fig — The intensity of the red or blue color in each row represents expression values, high or low respectively, which were scaled across both depots, and each column represents the culture condition (culture with 0, 1, 10, or 1000 nM Dex is designated Om0, Om1, Om10, Om1000, Abdsc0, Abdsc1, Abdsc10, Abdsc1000) as described in Methods. (TIF) [file pone.0167337.s001.tif]
